# Exhaled nitric oxide in early rheumatoid arthritis and effects of methotrexate treatment

**DOI:** 10.1038/s41598-022-10334-5

**Published:** 2022-04-20

**Authors:** Tomas Weitoft, Anders Lind, Anders Larsson, Johan Rönnelid, Marieann Högman

**Affiliations:** 1 Centre for Research and Development, Uppsala University/Region Gävleborg, 80187 Gävle, Sweden; 2 Department of Medical Science, Rheumatology, Uppsala University, 75185 Uppsala, Sweden; 3 Department of Medical Science, Clinical Chemistry, Uppsala University, 75185 Uppsala, Sweden; 4 Department of Immunology, Genetics and Pathology, Uppsala University, 75185 Uppsala, Sweden; 5 Department of Medical Science, Respiratory, Allergy and Sleep Research, Uppsala University, 75185 Uppsala, Sweden; 6 Department of Rheumatology, Region Gävleborg, Box 1512, 80139 Gävle, Sweden

**Keywords:** Rheumatic diseases, Inflammation

## Abstract

Patients with established rheumatoid arthritis (RA) and disease modifying treatments have lower nitric oxide (NO) levels in the alveolar compartment (C_A_NO) and in the airway wall (C_aw_NO), but also higher diffusion capacities for NO in the airways (D_aw_NO) compared to matched controls. The aim of the present study was to investigate the NO lung dynamics in patients with recent onset RA before and after immune suppression with methotrexate therapy. Patients with early RA and antibodies against anticitrullinated peptides (ACPA) were recruited. Measurement of exhaled NO and inflammatory markers in serum were performed. Clinical disease activity was evaluated with Disease Activity Score for 28 joints. Healthy individuals were used as matched controls. Data are presented as median (lower quartile, upper quartile) values. RA patients (n = 44) had lower exhaled NO (F_E_NO_50_) 16 (10–24) ppb compared to controls 21 (15, 29) ppb, p = 0.013. In NO-dynamics, C_A_NO was lower in RA patients 1.6 (1.0, 2.2) ppb compared to the control subjects 2.3 (1.3, 3.1) ppb, p = 0.007. C_aw_NO was also lower in the RA patients 55 (24, 106) ppb compared to control subjects 124 (110, 170) ppb, p < 0.001, but D_aw_NO was higher 17 (8, 30) mL/s and 9 (5, 11) mL/s respectively, p < 0.001. Methotrexate treatment for three months reduced disease activity, but did not change the NO dynamics. In conclusion, the altered NO dynamics of the lung in ACPA-positive RA patients are already present in the early stages of the disease before any treatments and do not change after methotrexate therapy suggesting a role in the pathogenesis.

## Introduction

Nitric oxide (NO) is an endogenous biatomic gaseous molecule involved in several important biological processes in the human body, with a key position in the inflammatory reaction by promoting vascular dilatation and permeability. NO is produced by many different cells via an oxidation of the amino acid L-arginine that uses nitric oxide synthase (NOS), and in this reaction the amino acid L-citrulline is also produced^1^. Small amounts of NO can be detected in the exhaled breath of healthy individuals. Elevated levels have been found in patients with inflammatory pulmonary diseases, e.g. asthma and chronic obstructive pulmonary disease (COPD) and also in rheumatic diseases, e.g. psoriatic arthritis, systemic sclerosis, Sjogren’s disease and systemic lupus erythematosus^2–7^.

The fraction of NO in exhaled gas (F_E_NO) depends mainly on the production of NO in the cells of the airways and alveoli and is governed by the diffusing capacity of NO in the airways. The NO dynamics of the lung can be estimated by using multiple NO measurements at different exhaled flows. A plot of NO output versus flow gives a non-linear curve that can be mathematically calculated. The Högman-Meriläinen algorithm (HMA) gives estimates of the alveolar concentration (C_A_NO), airway compartment diffusing capacity (D_aw_NO) and content of NO in the airway wall (C_aw_NO)^3^. These estimates are called NO parameters^8^.

Rheumatoid arthritis (RA) is a chronic autoimmune disease that can cause joint destruction, but other tissues may also be involved. In RA patients, there is an increase of endogenous NO production in the inflamed joints and higher levels are also found in the serum that correlates with disease activity^9,10^. NO has been shown to be a mediator of apoptosis in the synovial lining and articular cartilage of the rheumatoid joint^11,12^. Pulmonary changes are frequently found in RA patients that may be caused by the disease itself or the antirheumatic treatments^13^.

In approximately 70% of RA patients anti-citrullinated protein/peptide antibodies (ACPA) or rheumatoid factor (RF) are found, and these antibodies may be involved in the pathogenesis of the disease^14^. RA patients with autoantibodies have a more severe disease course than those without. Seropositive RA may therefore be a different disease entity than RA without detectable levels of ACPA or RF. Pulmonary or airway changes are often found in patients with early RA and also in ACPA-positive healthy individuals^15^. Theories for the pathogenesis of RA include inhaled exogenous factors, such as smoke or silica dust and/or other factors that activate the adaptive immune system in the airways that lead to ACPA and RF production^13,16^. The discovery of lymphocytic infiltration with germinal centres in bronchial biopsies from ACPA/RF-positive subjects without arthritis suggests a local ACPA production in the airway mucosa^15^.

In a previous study of RA patients with established disease and long-term anti-rheumatic treatments, we surprisingly found not only lower NO levels in the alveolar and airway compartments, but also higher diffusion capacities for NO in the airways compared to a matched control group of healthy individuals^17^. It was unclear if these findings were the effects of immunosuppressive treatment, a consequence of long-term chronic disease, or a part of the pathogenesis and disease process.

Therefore, the aim of the present study was to investigate the NO lung dynamics in patients with recent onset RA before the initiation of any disease-modifying antirheumatic drug.

(DMARD). In addition, we wanted to elucidate if these levels changed after three months of immunosuppressive treatment with methotrexate (MTX).

## Patients and methods

### Study design

#### Protocol A

All RA patients with serum positive for ACPA and meeting the 2010 ACR/EULAR criteria were recruited on their first visit to the Department of Rheumatology at Gävle hospital, Sweden^18^. Patient enrolment occurred March 2017 to November 2019. Patients with a symptom duration exceeding two years at the time of diagnosis, those taking > 10 mg prednisolone daily and those with difficulties understanding study information were excluded. Data from healthy, non-smoking subjects without atopy from a previous study were used as controls, and were matched for sex, age and body mass index (BMI)^19^.

#### Protocol B

Patients with active RA disease (DAS28 > 2.6) started treatment with MTX after the initial testing if there were no contraindications such as pregnancy, elevated liver enzymes or drug fear. The patients returned for a follow-up visit after 3 months. The same information as for the inclusion visit was collected.

### Data collection

The patients were evaluated clinically by their treating rheumatologist (TW, ALi). Disease activity was measured using the Modified Disease Activity Scores for 28 joints (DAS28) and disability with the Swedish version of the Stanford Health Assessment Questionnaire (HAQ)^20,21^. Measurements of exhaled NO and thereafter lung function were obtained together with a serum analysis for inflammatory markers.

#### NO analysis

In compliance with the 2005 American Thoracic Society and the European Respiratory Society (ERS) recommendations for NO measurements, the exhaled NO was analysed at an exhalation flow rate of 50 mL/s (F_E_NO_50_) using EcoMedics DLC 88 (Eco Medics AG, Dürnten, Switzerland)^22^. In compliance with the ERS technical standards of modelling NO dynamics, we used the nonlinear method with exhalation flows of 20, 100 and 300 mL/s^23^. NO free gas was inhaled and flow resistors facilitated a constant exhaled flow. The temperature was constant through all measures. A visual feedback system helped to guide the subjects so they could achieve the targeted flow throughout the exhalation. The non-linear HMA method was used with the software in the NO analyser. For quality control, a calculated F_E_NO_50_ for the HMA was derived for each subject and compared to the measured value. The HMA method estimates the following NO parameters: C_A_NO, C_aw_NO and D_aw_NO.

#### Lung function

Pre-bronchodilator spirometry lung function testing was performed using Welch Allyn. Spiro Perfect II (Welch Allyn, New York, USA). Results of forced expiratory volume at one second (FEV_1_) and forced vital capacity (FVC) are expressed as ratios and also as the percent of the predicted values based on age and sex according to Hedenström et al.^24,25^.

#### Blood analysis

Blood samples were collected for analysis of inflammatory markers, such as Erythrocyte Sedimentation Rate (ESR) and serum C-Reactive Protein (s-CRP). ACPA subclasses of IgA and IgG were analysed as anti-cyclic citrullinated peptide version 2 (anti-CCP2) and RF subclasses of IgA and IgM RF were analysed with fluorescence immunoassay (Elia, Thermo Fischer Scientific, Uppsala, Sweden) on a Phadia250 instrument (Thermo Fisher Scientific) according to the manufacturer’s instructions. All patients and all autoantibodies were investigated in parallel on one occasion. Cut-off levels for anti-CCP2 IgG and IgA were 7 arbitrary units. For RF IgM it was 9 arbitrary units and for IgA 5 arbitrary units. Serum nitrate/nitrite (NOx) was analysed using a Cayman nitrate/nitrite colorimetric assay kit (Ann Arbor, Michigan 48108 USA). The total coefficient of variation for the NOx assay was 3.4% and the limit of detection was 1 µM/L.

### Statistical analyses

All statistical analyses were performed using SPSS, v. 26 for Windows (SPSS Inc., Chicago, IL, USA). Data are expressed as median as well as lower and upper quartiles. The Mann–Whitney *U* test was used to make unpaired comparisons between any two groups. For pairwise comparisons the Wilcoxon signed rank test was applied. Correlations were tested with Spearman rank order correlation. A *p*-value of < 0.05 was considered significant.

### Ethical approval

The study was performed in accordance with Helsinki Declaration. The Regional Review Board in Uppsala, Sweden, approved the study on 7 October 2015 (Dnr 2015/370-1). All patients provided written informed consent.

## Results

**Figure 1:**
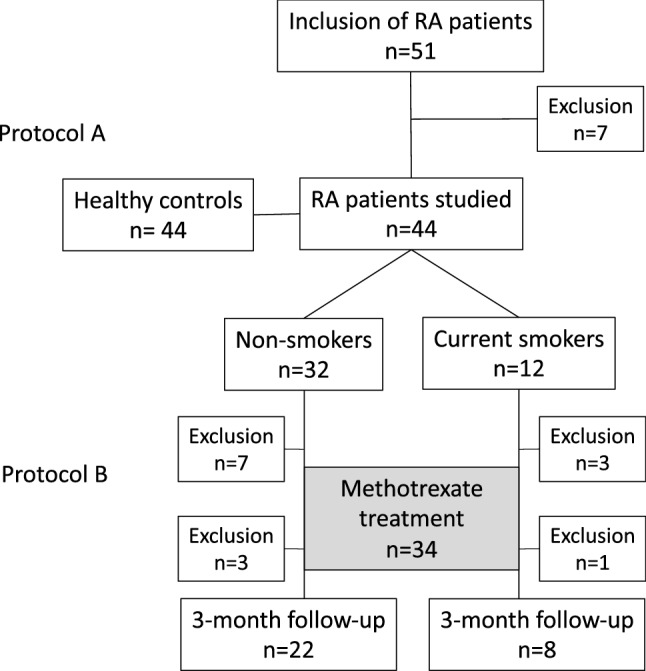
Flowchart of included and excluded patients in protocol A and B of the study.

A total of 51 patients were recruited to the study. All were ACPA positive and 80% of them were also RF positive. One patient was excluded because the criteria for RA diagnosis was not fulfilled. One patient who had too high daily dose of prednisolone was also excluded. Additionally, three more patients whose symptom duration at time of diagnosis was too long were excluded as were two patients who failed the exhale procedure. The remaining 44 participants were matched with healthy controls for the NO measurement comparisons (Fig. 1).

Co-morbidities found among the patients were chronic obstructive pulmonary disease (4 patients), hypertension (14 patients), diabetes mellitus (8 patients) and bronchial asthma (3 patients). Twelve patients (27%) were current smokers. Intra-articular glucocorticoids were administered to 14 patients during the inclusion visit.

### Protocol A

There were no statistically significant differences in regard to age, sex and BMI between the RA patients and matched control subjects. For the RA patients, the exhaled NO, F_E_NO_50_, as well as the NO parameters C_A_NO and C_aw_NO were lower, but the D_aw_NO was higher (Table 1). However, the control subjects were all non-smokers, and when the non-smoking RA subjects (n = 32) were compared to their matched controls there was no difference in F_E_NO_50_, but the C_A_NO and C_aw_NO were higher and the D_aw_NO was lower (Table 1). Non-smoking RA patients (n = 32) had higher values of F_E_NO_50_ 19 (13, 25) ppb and C_A_NO 1.9 (1.2, 2.3) ppb when compared to smoking RA patients (n = 12) who had 10 (5, 16) ppb F_E_NO_50_ (p = 0.002) and 1.0 (0.4, 1.3) ppb C_A_NO (p = 0.004). No differences were found in C_aw_NO 64 (33, 115) ppb and 24 (20, 75) ppb respectively (p = 0.086), and D_aw_NO 16 (9, 31) mL/s and 19 (7, 29) mL/s respectively (p = 0.809) (Fig. 1 Supplement).

**Table 1:** Characteristics of patients and control subjects in Protocol A. Data given in median (lower and upper quartiles).

	RA patients n = 44	Control subjects n = 44	p-value
Age (years)	62 (51, 72)	55 (50, 69)	0.193
Sex (% female)	59%	59%	1.0^1^
Symptom duration (months)	4 (2, 8)		
BMI	27 (24, 32)	25 (24, 28)	0.079
Current smoker (%)	12/44 (27%)	0 (0%)	0.001^1^
Non-smoker (%)	32 (73%)	44 (100%)	0.001^1^
DAS28	4.51 (3.68, 5.05)		
HAQ	0.88 (0.50, 1.25)		
*NO analysis*			
F_E_NO_50_ ppb	16 (10, 24)	21 (15, 29)	0.013
C_A_NO ppb	1.6 (1.0, 2.2)	2.3 (1.3, 3.1)	0.007
C_aw_NO ppb	55 (24, 106)	124 (110, 170)	< 0.001
D_aw_NO mL/s	17 (8, 30)	9 (5, 11)	< 0.001

	n = 32	n = 32	
**NO analysis from non-smoking subjects**			
F_E_NO_50_ ppb	19 (13, 25)	20 (15, 29)	0.413
C_A_NO ppb	1.9 (1.2, 2.3)	2.5 (1.5, 3.1)	0.019
C_aw_NO ppb	64 (33, 115)	129 (114, 171)	0.001
D_aw_NO mL/s	16 (9, 31)	7 (4, 11)	< 0.001

^1^Pearson Chi-square analysis.

BMI, body mass index; DAS28, disease activity score for 28 joints; HAQ, the Swedish version of the Stanford Health Assessment Questionnaire; F_E_NO_50_, fraction of exhaled nitric oxide at the flow of 50 mL/s; C_A_NO, alveolar nitric oxide; C_aw_NO, nitric oxide content in the airway wall; D_aw_NO, nitric oxide diffusion capacity over airway wall.

### Protocol B

Methotrexate treatment was started in 34 patients. From these, four did not attend the follow-up visit (two stopped therapy because of side effects, one was uncomfortable with the spirometry, and one did not show up due to the SARS-CoV-2 pandemic), Fig. 1. In the remaining 30 patients the median weekly MTX dose at follow-up was 20 (15, 20) mg.

After three months, the MTX-treated patients showed a reduced disease activity as evidenced by a reduction in their DAS28 scores and s-CRP levels. There was also an improvement in function according to their HAQ scores, Table 2. However, there were no differences between the non-smokers and current smokers (p = 0.661, p = 0.722, p = 0.097 respectively). Neither NOx, F_E_NO_50_ nor any of the NO parameters changed after three months of MTX treatment (Table 2). There were no statistically significant correlations between F_E_NO_50_ and NO parameters with NOx, DAS28, HAQ and s-CRP before or after treatment.

**Table 2:** Characteristics of smoking and non-smoking patients in Protocol B. Data given in median (lower and upper quartiles).

	Non-smokers n = 22			Current smokers n = 8		
	baseline	follow-up	p-value	baseline	follow-up	p-value
Age (years)	59 (49, 72)			58 (55, 71)		
Sex (% female)	64%			25%		
MTX	20 (15, 20)			20 (15, 20)		
DAS28	4.6 (3.5, 5.4)	2.5 (1.9, 3.2)	< 0.001	4.7 (4.2, 5.4)	2.8 (1.6, 3.3)	0.005
HAQ	1.0 (0.5, 1.3)	0.1 (0.0, 0.5)	< 0.001	1.0 (0.6, 1.3)	0.6 (0.1, 1.2)	0.036
**NO analysis**						
F_E_NO_50_ ppb	19 (12, 25)	21 (11, 24)	0.291	9 (5, 14)	7 (6, 8)	0.401
C_A_NO ppb	2.1 (1.1, 2.3)	1.8 (1.2, 2.1)	0.446	0.9 (0.3, 1.2)	1.1 (0.8, 1.1)	0.161
C_aw_NO ppb	65 (38, 119)	52 (24, 87)	0.328	21 (17, 61)	21 (14, 45)	0.398
D_aw_NO mL/s	16 (8, 31)	22 (12, 38)	0.929	17 (8, 27)	20 (10, 27)	0.612
**Lung function**						
FEV_1_/FVC	0.78 (0.73, 0.81)	0.78 (0.74, 0.81)	0.217	0.69 (0.62, 0.78)	0.67 (0.62, 0.79)	0.609
FVC-% predicted	85 (79, 97)	85 (74, 97)	0.948	78 (74, 87)	78 (72, 83)	0.779
FEV_1_-% predicted	91 (79, 99)	90 (77, 99)	0.745	79 (62, 85)	77 (62, 85)	0.484
**Blood analysis**						
s-CRP (mg/L)	8.2 (2.3, 23)	2.7 (1.1, 5.7)	0.003	7.4 (3.3, 18.5)	1.8 (1.1, 2.3)	0.005
NO_x_ (µmol/L)	2.4 (1.6, 3.2)	2.6 (2.3, 3.6)	0.054	1.7 (1.2, 2.5)	2.1 (1.7, 3.2)	0.237

MTX, methotrexate; DAS28, disease activity score for 28 joints; HAQ, the Swedish version of the Stanford Health Assessment Questionnaire; F_E_NO_50_, fraction of exhaled nitric oxide at the flow of 50 mL/s; C_A_NO, alveolar nitric oxide; C_aw_NO, nitric oxide content in the airway wall; D_aw_NO, nitric oxide diffusion capacity over airway wall; FEV_1_, forced expiratory volume at 1 s; FVC, forced vital capacity; s-CRP, serum C-reactive protein; NOx, nitrate/nitrite in serum.

## Discussion

In patients with recent onset ACPA positive RA the NO content in the airway wall was lower, NO diffusion capacity over the airway wall was higher and the alveolar NO was lower when compared to matched healthy controls. Excluding current smokers from the analysis gave comparable F_E_NO_50_ values, but C_aw_NO, D_aw_NO and C_A_NO still differed from the matched healthy controls. MTX treatment neither affected exhaled NO nor the serum nitrate/nitrite levels, although the patients showed a reduction in disease activity and disability.

The altered NO dynamics in the lungs of patients with recent onset RA, support the theory that the processes that lead to the manifestations of the disease may start in the lung or the mucosa of the airways^13^. It is well known that smoking is involved in the pathogenesis of RA and is associated with a subsequent high risk for developing ACPA-positive RA twice as high for male smokers and 1.3 times higher for female^26^. The low NO production in the lungs may have an important role in the RA pathogenesis. Low levels caused by the early disease process may be aggravated by smoking and together they may contribute to a more severe disease course. The processes that cause changes in the NO dynamics of non-smoking RA patients are not known. It has been proposed that other environmental exposures to the lung could interact with the disease progression and therefore nicotine is not the inducing component^13,27,28^.

In patients with early RA, the NO values from the extended NO analysis were different from those of their matched healthy controls. Our matched controls were non-smokers and 27% of our patients were current smokers. It is known that smoking tobacco reduces exhaled F_E_NO_50_ and it remains lower than healthy subjects even after smoking cessation^29,30^. This has been attributed to the inadequate supply of the cofactor tetrahydrobiopterin (BH4) needed for the NOS to be able to convert L-arginine to L-citrulline in the production of NO^29^. These in vitro experiments also showed that nicotine did not have a role in the inhibition of NOS.

When excluding smoking patients from the analysis we found no difference in F_E_NO_50_ compared to the control subjects. However, C_A_NO and C_aw_NO values were still lower with higher D_aw_NO. The main findings in this study and our previous study with treated RA patients are the low airway wall content of NO, C_aw_NO, and the high diffusing capacity for NO over the airway wall, D_aw_NO^17^. The findings that the levels of F_E_NO_50_ were the same in this study and our previous study when being compared to healthy subjects; can be explained by the two-compartment model of the NO lung dynamics. The model consists of the conducting airways and the gas exchange area. In the conducting airways, all airways of the lungs are equally represented. The gas exchange area contains the alveolar or acinar compartment as well as the respiratory bronchioles. When the alveolar gas with its low content of NO (C_A_NO) is expelled, the contribution of NO from the airways is driven by a concentration gradient of NO from the airway wall (C_aw_NO). The rate is governed by the diffusion capacity of the airway wall (D_aw_NO). Exhaled NO (F_E_NO_50_) is dependent on all of the NO parameters. Hence, a low C_aw_NO with a high D_aw_NO gave rise to a normal F_E_NO_50_ in our RA patients. Smoking subjects without RA have a normal D_aw_NO, which will therefore result in a low F_E_NO_50_^31^.

Anti-inflammatory therapy with oral glucocorticoids is often used in RA patients together with intra-articular glucocorticoid injections. The use of inhaled corticosteroids (ICS) in the treatment of asthma and COPD is widely accepted since they supress the inflammation in the airways and thereby lower the exhaled NO. Already after one day with ICS the F_E_NO_50_ is reduced, and it is the C_aw_NO that is responsible for the decrease since D_aw_NO is not affected by ICS^32,33^. In this study we looked at the MTX treatment and found that it was not causing any change in the NO dynamics of the lung. This is supported by the findings that MTX does not normalise the increased arginase activity in a rat model of RA^34^.

NO is produced by the conversion of L-arginine to L-citrulline by NOS. In health the endogenous synthesis of arginine is sufficient, but with increased metabolic requirements dietary supplementation is necessary; and consequently arginine is classified as a semi-essential amino acid. It was already discovered in the 1960s that arginine levels in RA patients were low^35^. One possible reason is the increased enzyme activity of arginase^36^. Chandrasekharan et al. have found an elevated arginase activity and signs of reduced NOS activity in the plasma of RA patients^37^. The authors suggest that as arginine is the most common substrate for both arginase and NOS, a substrate competition may reduce the NOS activity and diminish NO production. In addition, methylated arginine metabolites are potent inhibitors of NOS and may also block the cellular uptake of arginine^38^. A reduced NOS activity may explain the low levels found in the NO dynamics of our RA patients. Another possibility for this highly reactive NO gas to be low, is the rapid oxidization by reactive oxygen species (ROS) close to the site of NO production^39^. RA is a disease that is characterised by the formation of both ROS and reactive nitrogen species (RNS), which will cause the NO to be metabolized^40^. Further research is needed to discover the role of NO in RA.

There are limitations in our study. Firstly, conclusions are drawn from a limited number of RA patients. However, the results we see are in line with those from our previous study where the patients had an established RA, had been receiving long-term treatments and 63% of them were ACPA positive. Secondly, we did not have high resolution computer tomography to rule out any pulmonary abnormalities. This would have been of benefit in the interpretation of the NO analysis. A randomization to methotrexate or placebo in protocol B would have been ideal and the lack of a control group is another limitation.

## Conclusion

Our studies on the changes in the NO dynamics of patients with established and recent onset RA are the first to describe the higher diffusion capacity and lower levels in the airway wall and alveoli compared to matched control subjects. The changes appear early in the disease process and antirheumatic treatment with methotrexate has no impact. Today exhaled NO analysis have no place in the diagnosis or treatment of RA. Whether the reduced levels are present already in the pre-RA state and if immunological changes are associated is not known and should be studied in future research. These findings open a new window into the understanding of the pulmonary processes occurring during the different phases of the RA development.

## Supplementary Information

Supplementary Figure 1.

## Data Availability

Data are available upon reasonable request by contacting the corresponding author.

## Abbreviations

ACR/EULAR: American Congress of Rheumatology/European League Against Rheumatism
ACPA: Anti-citrullinated protein/peptide antibodies
Anti-CCP2: Anti-cyclic citrullinated peptide version 2
BMI: Body mass index
C_A_NO: Alveolar NO
C_aw_NO: NO content in airway wall
COPD: Chronic obstructive pulmonary disease
DAS28: Disease Activity Score for 28 joints
D_aw_NO: NO diffusion capacity over airway wall
DMARD: Disease-modifying antirheumatic drug
ESR: Erythrocyte sedimentation rate
F_E_NO_50_: Fraction of exhaled nitric oxide at the flow of 50 mL/s
FEV_1_: Forced expiratory volume at 1 s
FVC: Forced vital capacity
HAQ: Health assessment questionnaire
HMA: Högman Meriläinen algorithm
Ig: Immunoglobulin
MTX: Methotrexate
NO: Nitric oxide
NOS: Nitric oxides synthase
NOx: Nitrate/nitrite in serum
ppb: Parts per billion
RF: Rheumatoid factor
s-CRP: Serum C-reactive protein
