# Supplementary figures and images for: Exhaled nitric oxide in early rheumatoid arthritis and effects of methotrexate treatment
Source: Sci Rep. 2022 Apr 20;12:6489. doi: 10.1038/s41598-022-10334-5 (PMC9020158; doi:10.1038/s41598-022-10334-5)

Figure 1 (Supplement). NO dynamics at baseline in smoking and non smoking RA patients vs controls

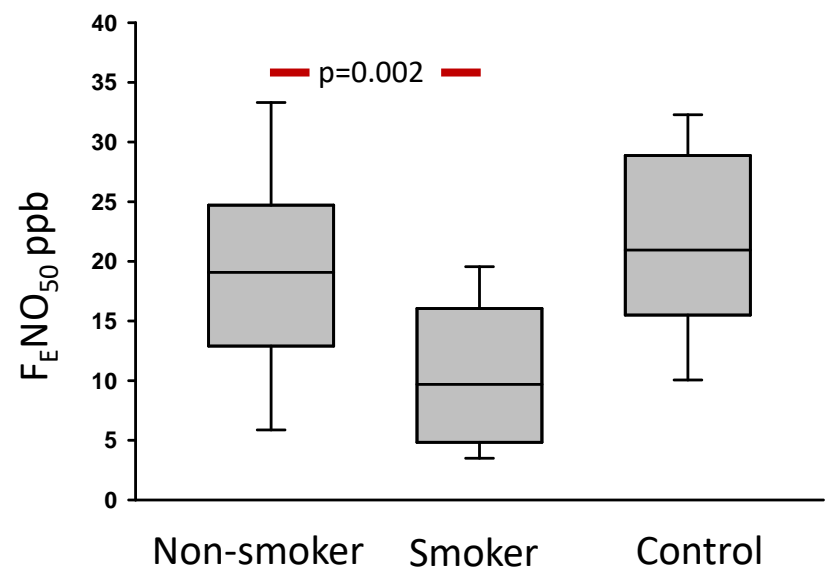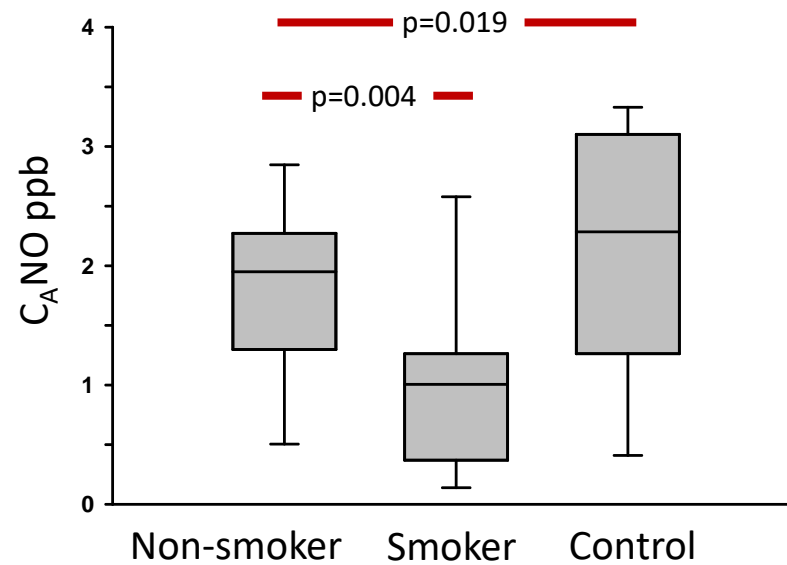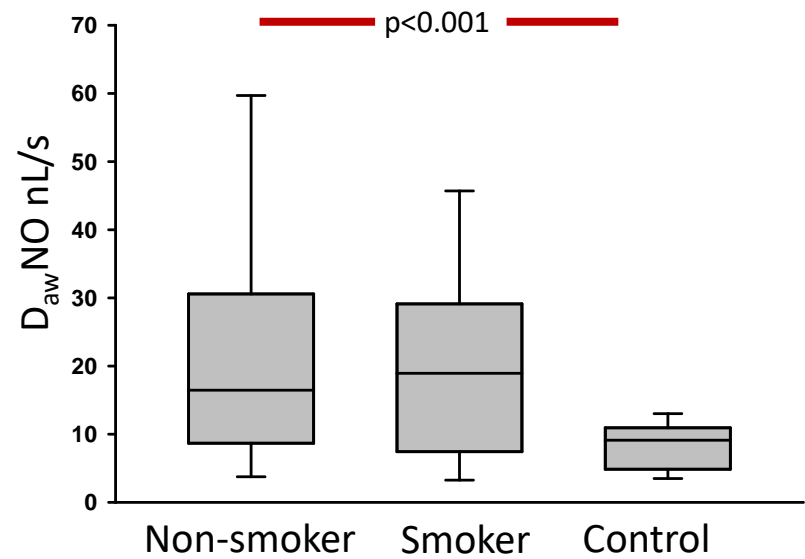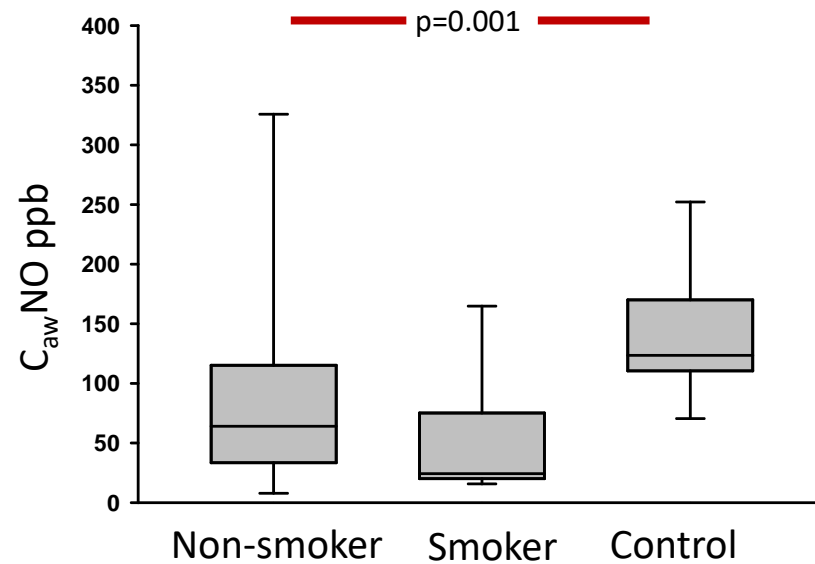

Supplement: Supplementary file 1 — Supplementary Figure 1. [file 41598_2022_10334_MOESM1_ESM.pdf]
